# Evaluating nanopore sequencing data processing pipelines for structural variation identification

**DOI:** 10.1186/s13059-019-1858-1

**Published:** 2019-11-14

**Authors:** Anbo Zhou, Timothy Lin, Jinchuan Xing

**Affiliations:** 1 Department of Genetics, Rutgers, the State University of New Jersey, Piscataway, NJ 08854 USA; 2 Human Genetics Institute of New Jersey, Rutgers, the State University of New Jersey, Piscataway, NJ 08854 USA

**Keywords:** Nanopore sequencing, Single-molecule sequencing, Structural variation, Pipeline evaluation

## Abstract

**Background:**

Structural variations (SVs) account for about 1% of the differences among human genomes and play a significant role in phenotypic variation and disease susceptibility. The emerging nanopore sequencing technology can generate long sequence reads and can potentially provide accurate SV identification. However, the tools for aligning long-read data and detecting SVs have not been thoroughly evaluated.

**Results:**

Using four nanopore datasets, including both empirical and simulated reads, we evaluate four alignment tools and three SV detection tools. We also evaluate the impact of sequencing depth on SV detection. Finally, we develop a machine learning approach to integrate call sets from multiple pipelines. Overall SV callers’ performance varies depending on the SV types. For an initial data assessment, we recommend using aligner minimap2 in combination with SV caller Sniffles because of their speed and relatively balanced performance. For detailed analysis, we recommend incorporating information from multiple call sets to improve the SV call performance.

**Conclusions:**

We present a workflow for evaluating aligners and SV callers for nanopore sequencing data and approaches for integrating multiple call sets. Our results indicate that additional optimizations are needed to improve SV detection accuracy and sensitivity, and an integrated call set can provide enhanced performance. The nanopore technology is improving, and the sequencing community is likely to grow accordingly. In turn, better benchmark call sets will be available to more accurately assess the performance of available tools and facilitate further tool development.

## Background

Structural variation (SV) is a major type of genomic variation. SVs are usually defined as genomic alterations that are larger than 50 bp in size and include insertions, deletions, duplications, inversions, and translocations. In humans, SVs account for the majority of the differences among individual genomes at the nucleotide level [1–3]. SVs have a profound impact on the genome architecture and are associated with a variety of diseases, including neurological diseases and cancer [4, 5]. Therefore, studying SVs and their functional implications is critical to understand the genomic architecture and the underlying genetic factors for many diseases.

DNA sequencing became one of the primary methods for SV identification in recent years [1–3]. Since 2005, a cost-effective, high-throughput generation of sequencing technology, termed next-generation sequencing, has been widely used in genomic research [6, 7]. However, for SV identification, the next-generation sequencing technology has its limitations due to its short read length (usually less than 200 bp), and most types of the evidence supporting an SV event are indirect (e.g., read depth, mismatch read pairs) [8].

The arrival of the third generation of sequencing technology, characterized by real-time, single DNA/RNA molecule sequencing, allows for much longer read lengths, opening new possibilities to address some of the limitations of next-generation sequencing for studying repetitive regions and SVs in the genome [3]. The nanopore sequencing technology commercialized by Oxford Nanopore Technologies (ONT) [9, 10] has gained popularity in recent years. Unlike many other sequencing methods, nanopore sequencing does not require the detection of a fluorophore which typically indicates a product of chemical or enzymatic reaction. Instead, single-stranded DNA/RNA molecules are directly sequenced by measuring the current disruption as a molecule passes through a nanopore [9]. Long reads obtained from the nanopore sequencing offer possibilities to detect SVs in a single continuous read instead of being inferred through indirect evidences from short reads. In the last several years, new computational tools have been developed specifically for long-read data and several studies have identified SVs using the nanopore data [11–14]. However, because the ONT sequencers were only recently launched, the tools available for aligning long-read data and detecting SVs have not yet been thoroughly evaluated.

In this study, we evaluated several aligners and SV callers on the nanopore data using four human nanopore datasets, including both empirical sequencing data and simulated reads. By comparing SV calls from seven aligner-SV caller combinations to established high-quality SV call sets, we evaluated the performance of long-read aligners, SV callers, and their overall combined performance. In addition, we developed a machine learning approach to integrate multiple SV call sets to produce a high-confidence call set.

## Result

### Selection of benchmarking dataset

For benchmarking, it is preferable to use several different datasets. In this study, we used four datasets: nanopore sequencing of the human samples NA12878 (referred to as NA12878 in the following text) and CHM13 (referred to as CHM13), simulated nanopore reads using the human genome assembly CHM1 (referred to as CHM1), and simulated nanopore reads using chromosome 20 of the human reference genome GRCh38 with artificially introduced SV events (referred to as Chr20).

NA12878 was sequenced at ~ 30× coverage by the nanopore whole-genome sequencing consortium [13]. For the corresponding SV true set, we used the SV call set generated by the Genome in a Bottle Consortium using the Pacific Biosciences (PacBio) platform [15]. CHM13 was a ~ 50× coverage whole-genome sequencing dataset of the CHM13hTERT human cell line on the Oxford Nanopore GridION [13]. The corresponding SV true set was generated using the PacBio platform with the SMRT-SV pipeline [16].

The CHM1 genome was assembled from a human haploid hydatidiform mole using reference-guided assembly [17]. Based on the CHM1 assembly, we simulated the nanopore sequencing reads to ~ 50× coverage (see the “Methods” section). Mapping the simulated nanopore reads resembles mapping empirical sequencing reads from an individual with a CHM1 genome. As a corresponding true SV call set for this sample, we used a SV call set generated using the PacBio platform [18].

The SV true sets for NA12878, CHM13, and CHM1 samples are dependent on their respective analysis pipelines and were filtered to select SVs with high accuracy. Therefore, it is likely that these true sets are incomplete which could affect the false-positive rate estimates for SV calling pipelines. To address this issue, we simulated chromosome 20 of the human reference genome GRCh38 with pre-defined SVs and generated nanopore sequencing reads at ~ 50× coverage for pipeline evaluation.

To assess the overall properties of the true sets, we collected several statistics of the true sets (Table 1). All true sets have more insertions than deletions. CHM1 and CHM13 true sets have more than twofold higher number of calls compared to the NA12878 set. SV size distribution analysis showed that most SVs are less than 500 bp in length (Additional file 1: Figure S1), and only a small number of SVs were larger than 10,000 bp (Additional file 1: Table S1, “true set”). For all sets, a peak could be observed at ~ 300 bp, an expected size for *Alu* transposable elements (Additional file 1: Figure S1).

**Table 1:** Summary statistics of the SV true sets

	NA12878 deletion	NA12878 insertion	CHM13 deletion	CHM13 insertion	CHM1 deletion	CHM1 insertion	Chr20 deletion	Chr20 insertion
SV count	4352	5783	10,671	20,497	10,784	15,158	96	181
Median size (bp)	312	300	304	318	69	103	318	296
Longest size (bp)	97,696	41,311	26,862	32,727	18,511	71,339	10,937	41,310
Shortest size (bp)	34	32	50	51	31	32	50	40

### Aligner and SV caller selection

Multiple aligners and SV callers were downloaded and tested on the nanopore datasets (Table 2, Additional file 1: Table S2). After initial testing, we excluded several tools from downstream analysis for a variety of reasons (see Additional file 1: Table S2 for details). As a result, we examined four aligners (minimap2, NGMLR, GraphMap, LAST) and three SV callers (Sniffles, NanoSV, Picky). We selected these tools based on their usability, compatibility, maintenance status, and popularity.

**Table 2:** Evaluated aligners and SV callers

Name	Type	Version	Release year	Threads	Language	Description	Citation
GraphMap	Aligner	0.5.2	2016	16	C++	Aligns nanopore long reads with circular genome handling	[19]
LAST	Aligner	941	2011	16	C++	Modified BLAST, outputs MAF format	[20]
minimap2	Aligner	2.1	2017	16	C	Aligns error-prone long reads, faster and more accurate than BWA	[21]
NGMLR	Aligner	0.2.6	2017	16	C++	Works with nanopore long reads to generate high-quality SV calls	[22]
NanoSV	SV caller	1.2.0	2017	16	Python	Identifies and clusters split reads based on genomic positions and orientations to identify breakpoint junctions of SVs	[23]
Picky	SV caller	0.2.a	2017	16	Perl	“Pick”-and-stitch segments from LAST alignments into representative alignments with a greedy algorithm	[24]
Sniffles	SV caller	1.0.8	2017	16	C++	Detects all types of SVs using split-read alignments, high-mismatch regions, and depth of coverage	[22]

### Aligner resource consumption and performance

**Fig. 1:**
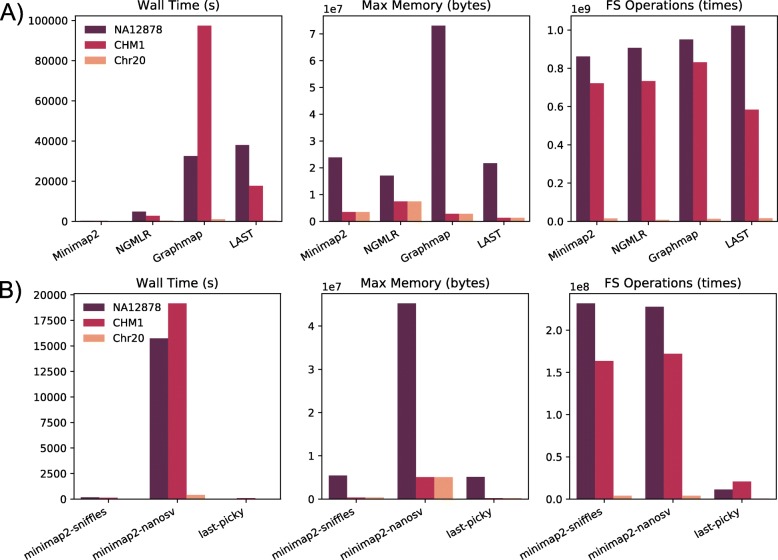
Resource consumption. **a** Aligner. **b** SV caller. The computing time (Wall Time), maximum memory usage (Max Memory), and file system operations (FS Operations) are shown. LAST’s Wall Time included Picky’s representative read selection step because Picky requires a specific output format from LAST in place of the default output. SV callers are noted with respect to the aligner used to map the reads. The CHM13 dataset was analyzed under multiple cluster configurations and therefore was not included in the computational resource evaluation

First, we compared the computational resource consumptions of the four aligners: minimap2, NGMLR, GraphMap, and LAST (Fig. 1a). Overall, each aligner performed similarly across datasets. Among the four aligners, minimap2 was the fastest by a large margin compared to other aligners, while GraphMap was the slowest. GraphMap also consumed the most memory. The file system operations were similar among all aligners (Fig. 1a, FS Operations). Next, we compared the quality of the aligned reads, such as the total mapped bases, mismatch rate, and genome coverage (Table 3). LAST’s output was not included in this analysis because its output was directly piped to the Picky for SV detection. Mapping coverage for NA12878 was ~ 24× for all aligners, compared to the raw sequencing coverage depth of ~ 30×. CHM13 had a higher coverage than NA12878, at ~ 42×. CHM13 also had a lower mismatch rate than NA12878, regardless of the aligner used. This difference might reflect the longer read length and the newer base-calling program used in the CHM13 dataset. The two simulated datasets, CHM1 and Chr20, have ~ 40× and ~ 50× coverage, respectively (Table 3).

**Table 3:** Alignment statistics

Aligner	Dataset	Bases mapped (Gb)	Mismatch rate	Coverage
minimap2	NA12878	77.5	1.97E−01	24.4
NGMLR	NA12878	73.6	1.92E−01	23.4
GraphMap	NA12878	80.2	2.17E−01	25.1
minimap2	CHM13	144.7	1.12E−01	43.7
NGMLR	CHM13	137.3	1.05E−01	42.0
GraphMap	CHM13	139.6	1.24E−01	42.7
minimap2	CHM1	128.6	1.35E−01	39.6
NGMLR	CHM1	127.6	1.35E−01	39.5
GraphMap	CHM1	130.4	1.52E−01	39.7
minimap2	Chr20	3.3	1.35E−01	48.5
NGMLR	Chr20	3.2	1.34E−01	47.4
GraphMap	Chr20	3.3	1.54E−01	49.1

### SV calling pipeline resource consumption and call set evaluation

Next, we compared computational resource consumption for three SV callers: NanoSV, Sniffles, and Picky (Fig. 1b). NanoSV and Sniffles results were collected based on the minimap2 alignment, and Picky results were based on the LAST alignment. Time and memory usage results highlighted that NanoSV consumed substantially more resources than the other two SV callers. The main time-consuming step of the NanoSV analysis was calculating the depth of coverage at the potential SV breakpoints. Picky performed fewer file system operations partially because the “select representative reads” step was already performed in combination with LAST before the SV calling step.

Because the overall mapped bases and coverages were similar for all aligners, we evaluated minimap2, NGMLR, and GraphMap as aligners in combination with Sniffles and NanoSV. The LAST alignment output format was not fully compatible with Sniffles and NanoSV, so we only evaluated LAST with Picky. LAST was chosen to run with Picky also because of its claimed synergy with Picky, and it was incorporated in the default Picky workflow [24]. In total, we tested seven SV calling pipelines: Minimap2-NanoSV, NGMLR-NanoSV, GraphMap-NanoSV, Minimap2-Sniffles, NGMLR-Sniffles, GraphMap-Sniffles, and LAST-Picky.

Each SV caller called different types of SVs with different abundance as shown in Additional file 1: Table S3. Deletion was the most abundant category, followed by insertion and duplication. The other categories, including inversion and translocation, all contained a small number of calls. Because only a small number of duplications were called and some SV true sets only contain insertions and deletions, the SV calls were grouped into two main categories: deletions and insertions (indels). As such, duplications were merged with insertions. The following analyses are performed on indels. Other types of SVs (e.g., inversions, translocations) from the call sets were not included in the evaluation.

**Fig. 2:**
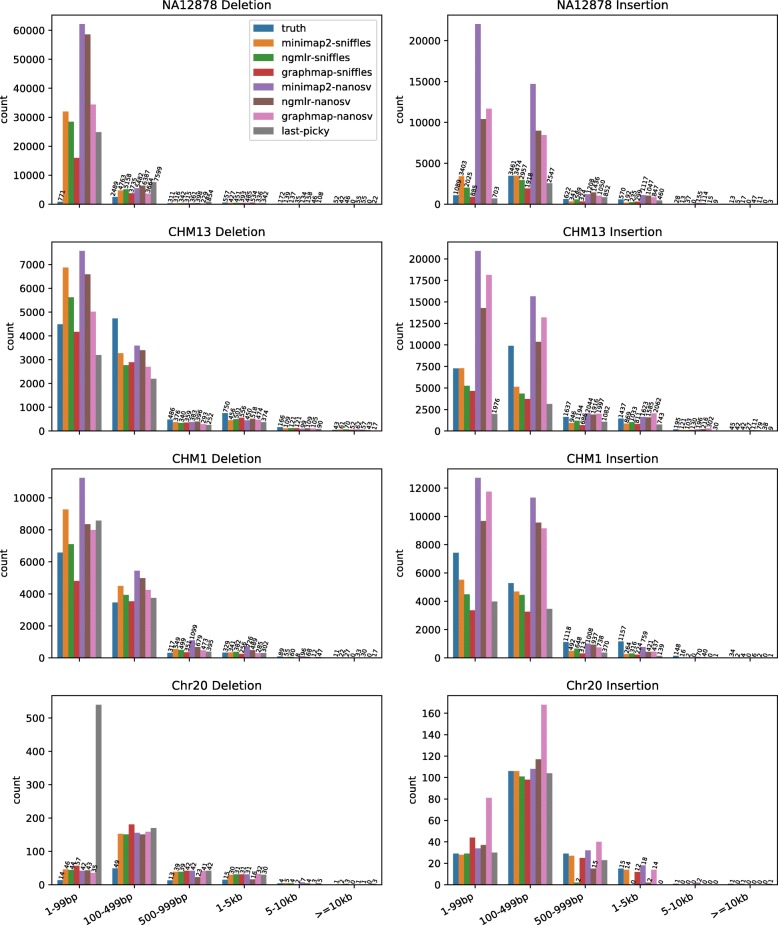
Insertion and deletion call set size distribution. The number of insertions and deletions in six size categories is shown for the true sets and calls from seven SV calling pipelines for the four datasets

The size distribution of the call sets showed more small indels than large indels, a pattern also observed among the true sets (Fig. 2, Additional file 1: Table S1). NanoSV called more insertions and deletions than Sniffles and Picky. In the simulated Chr20 dataset, Picky called more small deletions than any other pipeline. This is likely due to the Picky’s goal to maximize sensitivity and the high coverage of the Chr20 dataset resulted in a high false-positive rate.

**Fig. 3:**
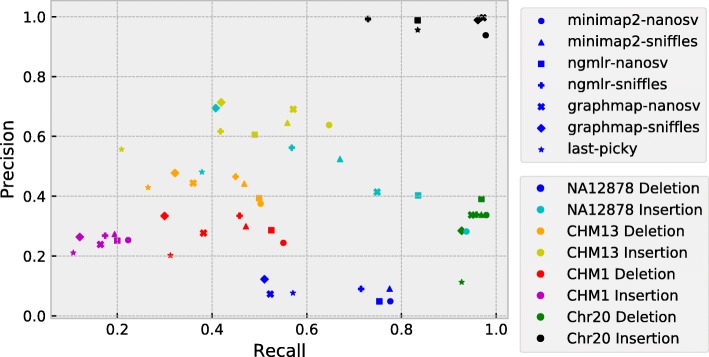
Precision-recall graph of SV calling pipelines. Pipelines are represented by shapes, and datasets are represented by colors as specified in the legend

To evaluate the quality of the indel calls, we calculated the precision, recall, and F1 score for each call set (Additional file 1: Table S1). The precision-recall graph showed that the four datasets occupy distinct areas (Fig. 3). The calls from the Chr20 dataset clustered on the right side of the plot, indicating that all call sets have high recall rates, although the precision was much higher for insertions than deletions. LAST-Picky deletion call set had the most false-positive calls (precision rate 11%), while NGMLR-Sniffles insertion calls had the lowest recall (73%). The NA12878 call sets, especially insertions (Fig. 3, cyan color), are in the central area of the graph and have the widest spread among different pipelines. The observed spread suggests that different pipelines had different precision versus recall advantages. As such, NanoSV call sets demonstrated highest recall rates (Fig. 3, cyan-colored circle, square, and cross), with Minimap2-NanoSV being the highest (Fig. 3, cyan-colored circle). Sniffles and Picky, on the other hand, had better precision rates, with the highest being GraphMap-Sniffles (Fig. 3, cyan-colored diamond). The CHM13 dataset clustered in the center area (Fig. 3, orange and yellow colors), suggesting different pipelines performed more consistent in this dataset. For CHM13, Minimap2-NanoSV had the highest recall rate and GraphMap-Sniffles had the highest precision. Finally, the CHM1 insertion call sets occupied the bottom-left area, which made it the worst call set given the true set, especially for the recall rates. CHM1 deletions were called with a small recall advantage over insertions (Fig. 3, red and magenta colors, respectively).

We next determined the rates of true-positive, false-negative, and false-positive calls in each call set stratified by indel size (Additional file 1: Figure S2). All pipelines performed the best for insertions in the Chr20 dataset, achieving a high-true positive rate (Additional file 1: Figure S2B). For deletions, all Chr20 call sets contained many false-positive calls, especially the LAST-Picky call set. Individual call datasets also showed different performance in different size distributions. In the NA12878 dataset, most pipelines identified many false-positive calls for SVs smaller than 200 bp, especially for deletions (Additional file 1: Figure S2). One possible reason for the high false-positive rates of the small SVs could be that nanopore sequencing reads have a high error rate at homopolymer and low-complexity regions. To test the effect of these repetitive regions, we subsequently excluded SVs overlapping simple repeats and low-complexity regions in the reference genome. The NA12878-filtered call sets indeed showed improvements for precisions, especially for deletions. However, filtering calls in the repetitive region also reduced the recall rates of the call sets (Additional file 1: Figure S3). For the CHM13 call sets, all pipelines generally had more false-negative calls when calling small SVs. CHM1 dataset displays a similar pattern to the CHM13 dataset, but showing a slightly lower true-positive rate, especially for insertions.

**Fig. 4:**
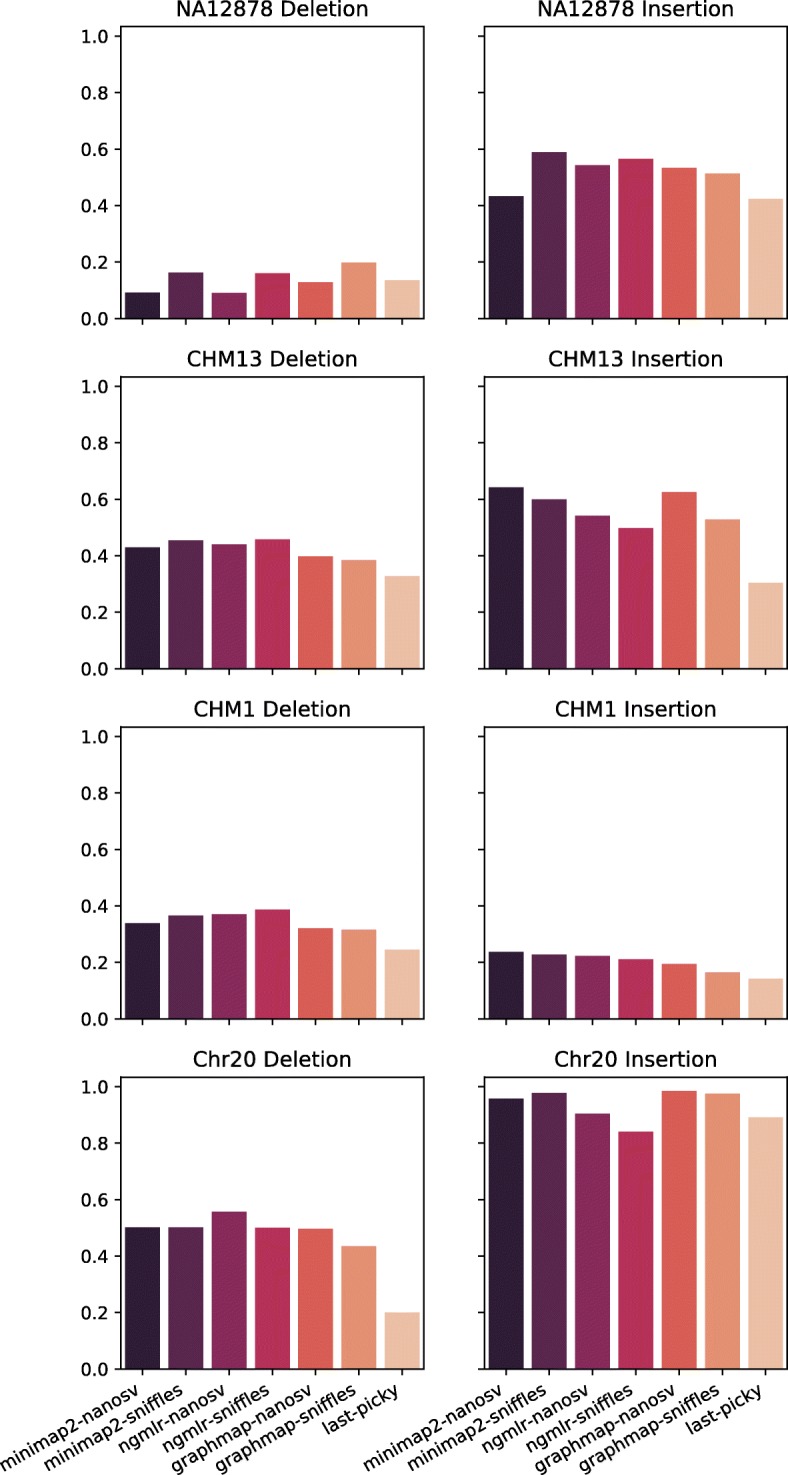
F1 scores for SV calling pipelines. F1 scores for the seven pipelines are shown for insertion and deletion calls of each dataset

To evaluate the overall performance of each pipeline and select the best pipeline, we calculated F1 score for insertions and deletions called by each pipeline in each dataset. F1 scores were comparable among all pipelines for a given dataset and SV type (i.e., insertion or deletion), but varied greatly among datasets and between insertion and deletion (Fig. 4, Additional file 1: Table S1). The best pipeline varied depending on the dataset and the type of SVs. Out of the eight dataset-SV type combinations, NanoSVs and Sniffles each had the highest F1 score in four combinations. In contrast, LAST-Picky had the lowest F1 scores in six combinations.

**Fig. 5:**
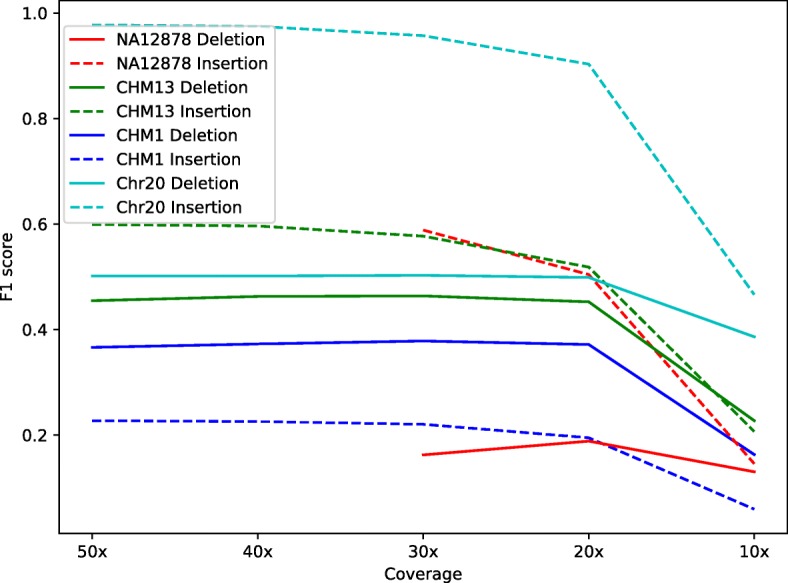
Impact of the sequencing coverage on the F1 score. For each dataset, subsampling was performed on the original dataset to generate lower coverage datasets. Solid line, deletions; dashed line, insertions. The data is based on the Minimap2-Sniffles pipeline

To evaluate the impact of the sequencing depth on indel calls, we created subsets of each dataset by randomly selecting reads to achieve 50×, 40×, 30×, 20×, or 10× sequencing coverages and calculated the F1 score of the Minimap2-Sniffles pipeline at different coverages (Fig. 5). In all datasets, F1 scores stayed relatively constant until 20× coverage and dropped dramatically at 10× coverage. One possible reason for the F1 score drop-off below 20× coverage could be that all SV callers apply a minimum number of supporting reads cutoff (e.g., we used 10 for Sniffles and Picky) and other quality requirements. Therefore, the coverage close to or lower than the cutoff would dramatically affect the performance of the callers.

### Consensus call set analysis and machine learning prediction

Next, we compared the SV calls among different pipelines. Overall, call sets from different pipelines each had many unique calls. As shown in the Venn diagrams of deletion calls in the NA12878 dataset, a large number of calls did not overlap between pipelines (Additional file 1: Figure S4). Even for pipelines using the same aligner or the same SV caller, the discrepancies remained large (Additional file 1: Figure S4).

**Fig. 6:**
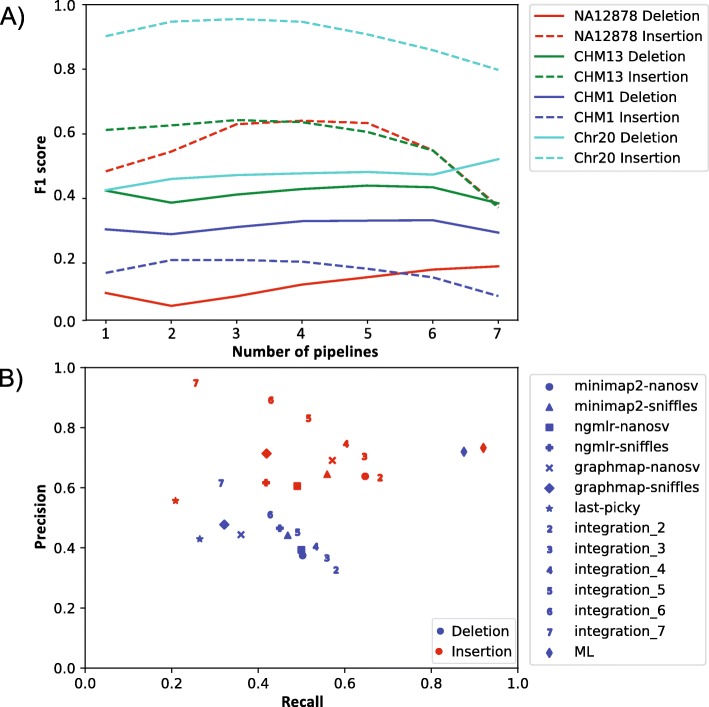
SV call set integration. **a** Consensus approach. Solid line, deletions; dashed line, insertions. F1 scores for insertions and deletions identified by a specified number of pipelines are shown. **b** Precision-recall graph of call sets from SV calling pipelines and integration approaches. Blue, deletions; red, insertions. Results from individual pipelines and the machine learning approach are represented by shapes as specified in the legend. Results from the consensus approach are represented by numbers. For example, “2” represents the consensus call set from two callers

The large proportion of unique calls in each pipeline suggested there is a potential to improve SV calling quality by integrating calls from multiple pipelines. To evaluate the effect of integrating multiple call sets, we merged all call sets for each dataset, while tracking the number of call sets for each merged SV call. For deletions, requiring evidence from multiple pipelines improved the F1 scores of the call sets (Fig. 6a). The F1 scores for deletions in all four datasets reached a peak when requiring overlaps of six or seven pipelines. For insertions, applying the consensus pipeline filter also increased the F1 scores, and calls shared among two or three pipelines resulted in the best F1 scores (Fig. 6a).

Overall, selecting calls supported by multiple pipelines showed improvement of F1 scores, but the improvement patterns were not consistent. Thus, we applied a more sophisticated call set integration approach by training a machine learning model based on the random forest algorithm. We selected seven SV features provided in the output of the SV callers, such as SV length, number of supporting reads, mapping quality, and confidence interval of the breakpoint (Table 4). Using the CHM13 dataset as a test set, we achieved F1 scores of 0.79 for deletions and 0.81 for insertions, a substantial improvement over the best simple integration method (0.47 for deletion and 0.67 for insertion). Unlike the simple integration method, the machine learning approach was able to improve recall rate without sacrificing the precision (Fig. 6b). Among the seven features, the most important contributing feature was SV length, which accounted for ~ 50% of the evidence, followed by the depth *P* value, read support, and mapping quality (Table 4). Similar to CHM13, the machine learning approach also produced improvement for most other data sets (Additional file 1: Table S4). Because the depth *P* value is only provided by NanoSV, while the read support was provided by Sniffles and Picky (Table 4), the machine learning approach allowed us to consider additional information provided by different callers to produce a high-confidence call set.

**Table 4:** SV features and their contributions in the random forest classifier for CHM13

Feature	Description	SV caller			Contribution	
		Sniffles	NanoSV	Picky	Deletion (%)	Insertion (%)
SVLEN	Length of the SV	Yes	Yes	Yes	52	55
DEPTHPVAL	*P* value of the significance test of the depth of coverage at possible breakpoint junctions	No	Yes	No	20	15
RE	Read support	Yes	No	Yes	7	14
MAPQ	Median mapping quality of read pairs	Yes	Yes	No	10	8
CIEND	Confidence interval around the END position	No	Yes	Yes	3	3
CIPOS	Confidence interval around the POS position	No	Yes	Yes	2	2
PRECISE	Precise structural variant	Yes	Yes	Yes	5	1

“Yes/No” under SV callers indicates whether a feature is provided by an SV caller

## Discussion

Improvements in our ability to detect and evaluate SVs in the genome are crucial to improve our understanding of the functional impact of SVs. While next-generation sequencing technologies have revolutionized genomics, their short read length has hindered the ability to reliably detect SVs. Recently, ONT released its nanopore-based sequencers that are capable of generating long reads, potentially improving our ability to detect SVs. Using public high-coverage nanopore sequencing data and simulated data, we evaluated multiple aligners and SV callers to assess SV identification performance using nanopore long-read sequencing data.

We benchmarked four aligners: an older and established aligner LAST and three more recently developed long-read aligners (minimap2, NGMLR, and GraphMap). Alignment time and memory usage varied widely between the four aligners while differences with respect to the mapped reads were moderate. Minimap2 was the fastest aligner tested with the most mapped bases. Therefore, we recommend minimap2 as a default aligner for general use. Unlike the newer aligners, which output the alignments in Sequence Alignment Map (SAM) format, LAST uses Multiple Alignment Format (MAF) format. Although we tested converting the MAF format to SAM format, the resulting alignments are not fully compatible with SV callers expecting a SAM format input (data not shown). Therefore, we only evaluated the LAST-Picky pipeline.

The SV call sets differed dramatically among the pipelines, for both deletions and insertions. Unless the user is limited by specific requirements for SV calling, we recommend using minimap2 paired with Sniffles for the initial assessment of the data. This combination of tools showed the fastest processing time and a balanced overall performance in detecting both deletions and insertions. Our results are similar to a recent study on a different human sample [12]. On the other hand, for a specific project, the choice of the pipeline could depend on the need of the user for either high recall rate or high precision. Sniffles call sets showed the highest precision for most of the datasets tested, while NanoSV call sets generally had a higher recall rate, largely attributed to the higher number of SVs identified by NanoSV. Therefore, Sniffles should be used when high precision is the priority, while NanoSV should be considered if high sensitivity is desired and additional false-positive calls can be tolerated.

All four datasets we used in this study have their own advantages and limitations for SV caller evaluation. For the Chr20 simulation dataset, we incorporated SVs based on the SV distribution from a real call set and used empirical error profile from an ONT sequencing run to simulate reads that resemble a true human sample. The advantage of such a simulated dataset is that we know the true SVs that can be used to evaluate different pipelines. Nevertheless, the simulated reads are based solely on chromosome 20 and are unlikely to capture the true heterogeneity of the entire human genome. This could in part explain the better performance of the Chr20 call sets compared to call sets from the other three datasets. For the NA12878, the CHM13, and the CHM1 genome, we evaluated our SV calls against high-coverage datasets (40–60× coverage) generated using the PacBio sequencing technology [15, 18]. These three datasets are among the few available long-read datasets that attempt to produce high-confidence SV calls by employing several different SV calling pipelines and the de novo assembly approach. Although SV calls in the three PacBio datasets are likely to have a high accuracy, these datasets are limited in several ways. For example, some of the benchmark datasets only include deletions and insertions, whereas SV callers we employed also generated other types of SV calls. In addition, these datasets are based on the PacBio sequencing platform, which has its own limitations in terms of both sequencing technology and analysis tools. For example, one of the SV callers used to generate the benchmark, PBHoney [25], is an older SV caller and it is not actively maintained at the moment. Indeed, the vast majority of NA12878 deletions that are called by all seven pipelines were absent from the SV true set. One such deletion region is chr1:117,029,131-117,029,278, for which minimap2 alignment shows multiple nanopore sequencing reads with evidence of a deletion, while the PacBio BLASR alignment showed only low-quality alignments in the region (i.e., with a large number of mismatches) (Additional file 1: Figure S5). Therefore, some of these SVs are likely to be real in the nanopore data but false negative in the benchmark set. As long-read sequencing technology matures, more comprehensive true SV call sets will become available and improve the evaluation. More importantly, experimental validation of some SV calls is necessary to empirically assess the accuracy of the calls.

With the different datasets, we also assessed the impact of genome coverage on the SV identification among the SV callers. We sought to determine the minimum depth of coverage required to obtain a reasonable SV calling quality, given the limitation of budget and computational resources in research projects. For all three datasets, 20× coverage appeared to be the minimum coverage required to maintain the performance of the tools as judged by the F1 score. Given both the sequencing technology and the computational tools are under active development, we expect the coverage requirement will also be reduced in the future.

The SV calling results from the pipelines tested here showed that there is room for improvement for the tools in terms of both recall and precision. In the meantime, one potential way to improve the performance of the currently available SV callers is to use an integrative approach and combine calls from multiple pipelines. We evaluated the integration principle using two approaches: one simple consensus approach and one machine learning approach using the random forest algorithm that uses seven features from the SV caller outputs. Our results showed that both approaches can improve the F1 scores of the call sets. However, when combining the quality features provided by multiple call sets, the machine learning approach provided a much better overall performance compared to the simple consensus approach (Fig. 6b). This result suggests that when a true set is available for training, a machine learning approach can be a good way to produce high-quality call set from multiple callers. In general, these results demonstrated the value of an integrative approach and further supported the need for the systematic evaluation and development of integrative approaches. Several SV integration tools with a more sophisticated integration algorithm, such as MetaSV [26], svclassify [27], and Parliament [28], have been developed for integrating SV calling results from multiple sequencing technologies and SV callers, including single-molecule sequencing technologies. A similar algorithm can be applied to single-molecular sequencing SV callers and generate a high-quality consensus SV call set.

## Conclusions

Nanopore sequencing is a rapidly developing technology in terms of both sequencing technology and data analysis. For SV analysis, several new aligners and SV callers have been developed to leverage the long-read sequencing data. In addition, assembly-based approaches can also be used for SV identification. We have established a workflow for evaluating mappers and SV callers. We found that SV callers’ performance diverges between SV types. Therefore, our recommendations are tailored to the specific applications. For an initial analysis, we recommend minimap2 and Sniffles due to their high speed and relatively balanced performance calling both insertions and deletions. For more detailed analysis, we recommend running multiple tools and integrating their results for the best performance. When a high-quality true set can be defined, a machine learning approach, such as the one we proposed here, can be used to further improve the call set. Most analysis tools for nanopore sequencing are recently developed, and both accuracy and sensitivity can be improved. We expect resources from ONT and the nanopore sequencing community to accumulate as the technology improves and its user base grows. With more data being generated, better benchmark call sets will be available to more accurately assess the tool performance and facilitate future tool development.

## Methods

### Data set generation

The nanopore sequencing data of NA12878 in FASTQ format was obtained from the release 3 of the nanopore whole-genome sequencing consortium repository (https://github.com/nanopore-wgs-consortium/NA12878/blob/master/nanopore-human-genome/rel_3_4.md) [13]. The data was sequenced on the Oxford Nanopore MinION using 1D ligation kit. The SV call set for NA12878 was downloaded from ftp://ftp-trace.ncbi.nlm.nih.gov/giab/ftp/data/NA12878/NA12878_PacBio_MtSinai/NA12878.sorted.vcf.gz [15]. This call set was based on the whole-genome sequencing data of NA12878 at about 44× coverage using the PacBio platform. The SV call set was generated using three SV detection methods, including a local assembly pipeline [18]. Only SV calls with a “PASS” flag in the “FILTER” field was included in the analysis. This dataset was lifted over from human reference genome GRCh37 to GRCh38 using liftOver (https://genome.ucsc.edu/cgi-bin/hgLiftOver).

The CHM13 genome nanopore sequencing reads were downloaded from the release 2 of the nanopore whole-genome sequencing consortium (https://s3.amazonaws.com/nanopore-human-wgs/chm13/nanopore/rel2/rel2.fastq.gz). The SV calls were obtained from dbVar (ftp://ftp.ncbi.nlm.nih.gov/pub/dbVar/data/Homo_sapiens/by_study/vcf/nstd137.GRCh38.variant_call.vcf.gz).

The CHM1 genome assembly was downloaded from NCBI (ftp://ftp.ncbi.nlm.nih.gov/genomes/all/GCA/000/306/695/GCA_000306695.2_CHM1_1.1/GCA_000306695.2_CHM1_1.1_genomic.fna.gz). The nanopore sequence reads were simulated from the CHM1 assembly using NanoSim (ver 2.1.0) [29]. To generate a training dataset for nanopore sequencing read profile, DNA sample of the individual HuRef [30] was purchased from Coriell (NS12911, Camden, NJ, USA). The HuRef sample was sequenced in our lab to about 1× coverage with an ONT MinION sequencer (Additional file 1: Supplemental Text: HuRef Sequencing). The sequencing reads were then used to generate the read profile by NanoSim *read_analysis.py* command [29]. Using the read profile and the CHM1 genome as the input, NanoSim *simulator.py* command simulated in silico reads to about 50× target coverage (50,000,000 sequences) from the CHM1 genome. A high-quality SV dataset for CHM1 was generated using the PacBio technology by the local assembly approach [18]. This data was downloaded from http://eichlerlab.gs.washington.edu/publications/chm1-structural-variation/data/GRCh37/insertions.bed, and http://eichlerlab.gs.washington.edu/publications/chm1-structural-variation/data/GRCh37/deletions.bed. The dataset was lifted over from GRCh37 to GRCh38 using liftOver.

The R package RSVSim (ver. 1.24.0) [31] was used to simulate deletions and insertions in chromosome 20 of the human reference genome GRCh38. The number and size of each simulated SV were set to be identical to the NA12878 true set above (181 insertions and 96 deletions on chromosome 20). NanoSim was used to simulate reads to about 50× target coverage (1,200,000 reads) based on the same read profile trained by the HuRef reads.

### Read mapping and SV identification

The aligners and SV callers (Table 2) were downloaded and compiled on a high-performance computing cluster based on the Ubuntu 14.04 system. Each node has 2 AMD Opteron 6272 2.1 GHz 16-core processors and 256 Gb RAM. The CHM13 dataset contains a large number of long reads (e.g., more than 500,000 kb) that caused long-running time for some aligners. To optimize the alignment performance for CHM13, reads longer than 500 kb in length were excluded from the dataset when an alignment program stalled. For running LAST on the CHM13 dataset, reads that are larger than 300 kb were filtered out, and 39,911 reads that consistently caused memory shortages were excluded. The CHM13 dataset was analyzed under multiple cluster configurations and therefore was not included in the computational resource evaluation. The computational resource consumptions were recorded using GNU command “/usr/bin/time –v.” The depth of coverage of an alignment file was calculated by SAMtools *depth* command (ver. 1.6) [32]. The percentage of mapped reads, number of mapped bases, and mismatch rate of an alignment file were calculated by SAMtools *stats* command (ver. 1.6).

Evaluation of insertions and deletion call sets for each dataset was performed using BEDTools (ver. 2.27.1) [33]. Deletions were compared with the SV true sets using BEDTools *intersect* command requiring at least 50% overlap between the two regions. Because insertions were represented by a single base pair position in the reference genome, insertions were compared with the SV true sets using BEDTools *window* command where two insertions were considered an overlap if they were within 100 bp of each other. Precision rate, recall rate, and F1 score were calculated for each SV call set against their respective SV true set. Plots were generated using the matplotlib and seaborn library in Python3.

### Call set filtering

For both true sets and call sets, several filtering and processing steps were performed to generate comparable datasets. First, SV calls from unincorporated contigs and the mitochondrial genome were filtered out to generate call sets for SVs on autosomes (chromosomes 1–22), chromosome X, and chromosome Y. In each call set, insertions, duplications, and deletions were selected. Insertion and duplication calls were combined as one category (referred to as “insertions”) for comparison. SVs were then filtered for size between 30 and 100,000 bp. The resulted SV calls were sorted using BEDTools *sort* command and merged using BEDTools *merge* command.

### Coverage analysis

Random subsampling of the FASTA files in each analysis was performed using the seqtk toolset (https://github.com/lh3/seqtk) based on the minimum number of reads needed to reach an expected coverage depth ranging from 10× to each dataset’s original coverage, increasing by 10× each time. Subsampled reads at each coverage depth were mapped by minimap2, and SVs were called by Sniffles. The call sets were evaluated with the respective SV true set, and F1 score was calculated for each coverage depth in each comparison category.

### Consensus call set

To generate a consensus call set for each dataset, call sets from all pipelines for each dataset were concatenated to a single file. BEDTools *merge* function [33] was then used to merge the concatenated calls into a consensus call set. The number of pipelines identified each consensus SV was stored. The consensus SVs were then filtered based on the number of pipelines that identified them, ranging from two to seven, and compared to their respective true sets.

### Random forest classifier

SV calls from all seven pipelines for each pipeline were combined and labeled “true” or “false” based on whether they overlapped with the corresponding true set. The combined call set was randomly split into a training set (20% of the calls) and a testing set (80% of the calls) using the python package scikit-learn (v0.21.3, parameter “train_size=0.2”). The labeled SVs were learned and predicted by XGBoost (v0.90) random forest classifier [34] using the features selected from the “INFO” tag in the VCF files (Table 4). Precision and recall rate of the predictions were calculated by scikit-learn metrics.

## Supplementary information

**Additional file 1. **Supplementary Text: HuRef Sequencing. **Figure S1**. True set indel size distribution. **Figure S2**. Quality of each SV call set by size. **Figure S3**. Precision-recall graph of NA12878 SV calls before and after filtering repetitive genomic regions. **Figure S4**. SV call differences between pipelines. **Figure S5**. Nanopore and PacBio sequencing alignments comparison at an SV region. **Table S1**. SV call set evaluation. **Table S2**. Aligners and SV callers excluded from the analysis. **Table S3**. Counts of different types of NA12878 SVs called by the seven pipelines. **Table S4**. Statistics of random forest classifier on all datasets.

**Additional file 2.** Review history.

## Data Availability

The HuRef sequencing reads are available at the Rutgers University Community Repository (10.7282/t3-zw94-js46). The FASTQ format sequencing reads of the NA12878 data set is downloaded from the nanopore whole-genome sequencing consortium GitHub repository (https://github.com/nanopore-wgs-consortium/NA12878/blob/master/nanopore-human-genome/rel_3_4.md) [13]. The SV calls are download from the “Genome in a Bottle” FTP site (ftp://ftp-trace.ncbi.nlm.nih.gov/giab/ftp/data/NA12878/NA12878_PacBio_MtSinai/NA12878.sorted.vcf.gz) [15]. The CHM13 genome sequencing reads were downloaded from the nanopore whole-genome sequencing consortium (https://s3.amazonaws.com/nanopore-human-wgs/chm13/nanopore/rel2/rel2.fastq.gz) [35]. The SV calls for CHM13 were obtained from the dbVar FTP site (ftp://ftp.ncbi.nlm.nih.gov/pub/dbVar/data/Homo_sapiens/by_study/vcf/nstd137.GRCh38.variant_call.vcf.gz). The CHM1 genome assembly was downloaded from NCBI under accession number GCA_000306695.2 (ftp://ftp.ncbi.nlm.nih.gov/genomes/all/GCA/000/306/695/GCA_000306695.2_CHM1_1.1/GCA_000306695.2_CHM1_1.1_genomic.fna.gz). These SV calls were downloaded from the Eichler Lab website (http://eichlerlab.gs.washington.edu/publications/chm1-structural-variation/data/GRCh37/insertions.bed, http://eichlerlab.gs.washington.edu/publications/chm1-structural-variation/data/GRCh37/deletions.bed) [18]. The raw outputs for all pipelines on the four datasets and the data for chromosome 20 simulation are available in the Rutgers University Community Repository (10.7282/t3-zw94-js46). The codes used in the study, as well as a singularity package containing pre-installed programs and the seven pipelines, are available in the GitHub repository, at https://github.com/JXing-Lab/nanopore-sv-evaluation [36], and in the Zenodo repository, at https://zenodo.org/badge/latestdoi/187123521, under the open source MIT license.
