# Supplementary material for: Evaluating nanopore sequencing data processing pipelines for structural variation identification
Source: Genome Biol. 2019 Nov 14;20:237. doi: 10.1186/s13059-019-1858-1 (PMC6857234; doi:10.1186/s13059-019-1858-1)
Supplement: Supplementary file 1 — Additional file 1. Supplementary Text: HuRef Sequencing. Figure S1. True set indel size distribution. Figure S2. Quality of each SV call set by size. Figure S3. Precision-recall graph of NA12878 SV calls before and after filtering repetitive genomic regions. Figure S4. SV call differences between pipelines. Figure S5. Nanopore and PacBio sequencing alignments comparison at an SV region. Table S1. SV call set evaluation. Table S2. Aligners and SV callers excluded from the analysis. Table S3. Counts of different types of NA12878 SVs called by the seven pipelines. Table S4. Statistics of random forest classifier on all datasets. [file 13059_2019_1858_MOESM1_ESM.pdf]

Supplementary Material

for

Evaluating nanopore sequencing data processing pipelines for structure variation identification

Anbo Zhou, Timothy Lin, and Jinchuan Xing

Contents

Contents 1

HuRef Sequencing...2

Fig. S1: True set indel size distribution. 3

Fig. S2: Quality of each SV call set by size...4

Fig. S3: Precision-recall graph of NA12878 SV calls before and after filtering repetitive genomic regions. 6

Fig. S4: SV call differences between pipelines 7

Fig. S5: Nanopore and PacBio sequencing alignments comparison at an SV region...8

Table S1: SV call set evaluation. 9

Table S2: Aligners and SV callers excluded from the analysis. 13

Table S3: Counts of different types of NA12878 SVs called by the seven pipelines..13

Table S4: Statistics of random forest classifier on all datasets. 14

References 15

HuRef Sequencing

DNA sample of individual NS12911 was purchased from Coriell (Camden, NJ, USA). An input of 1.5 ug genomic DNA was sheared using a Covaris g-Tube (520079, Covaris, Woburn, MA, USA) to obtain 10 kb fragments. Starting with the fragmented DNA the sequencing library was constructed following the ONT protocol for 1D Genomic DNA by ligation kit (SQK-LSK108, Oxford Nanopore Technologies, Oxford, UK). The library was sequenced on ONT MinION using a flow cell FLO-MIN106D (Oxford Nanopore Technologies, Oxford, UK). Data from the sequencing run was basecalled with Albacore (Ver. 2.0.2, Oxford Nanopore Technologies, Oxford, UK). The sequencing run generated 1,026,451 reads and 2,985,936,734 bps. The median read length for the sequencing run was 1,418 bps with a mean q score of 8.6. The data is available at https://doi.org/doi:10.7282/t3-zw94-js46.

Fig. S1: True set indel size distribution. The size distribution of insertions and deletions in the four SV true sets are shown. The indels were filtered to remove the top 10% largest calls to improve the visibility. Counts of larger SVs are listed in Table S1.

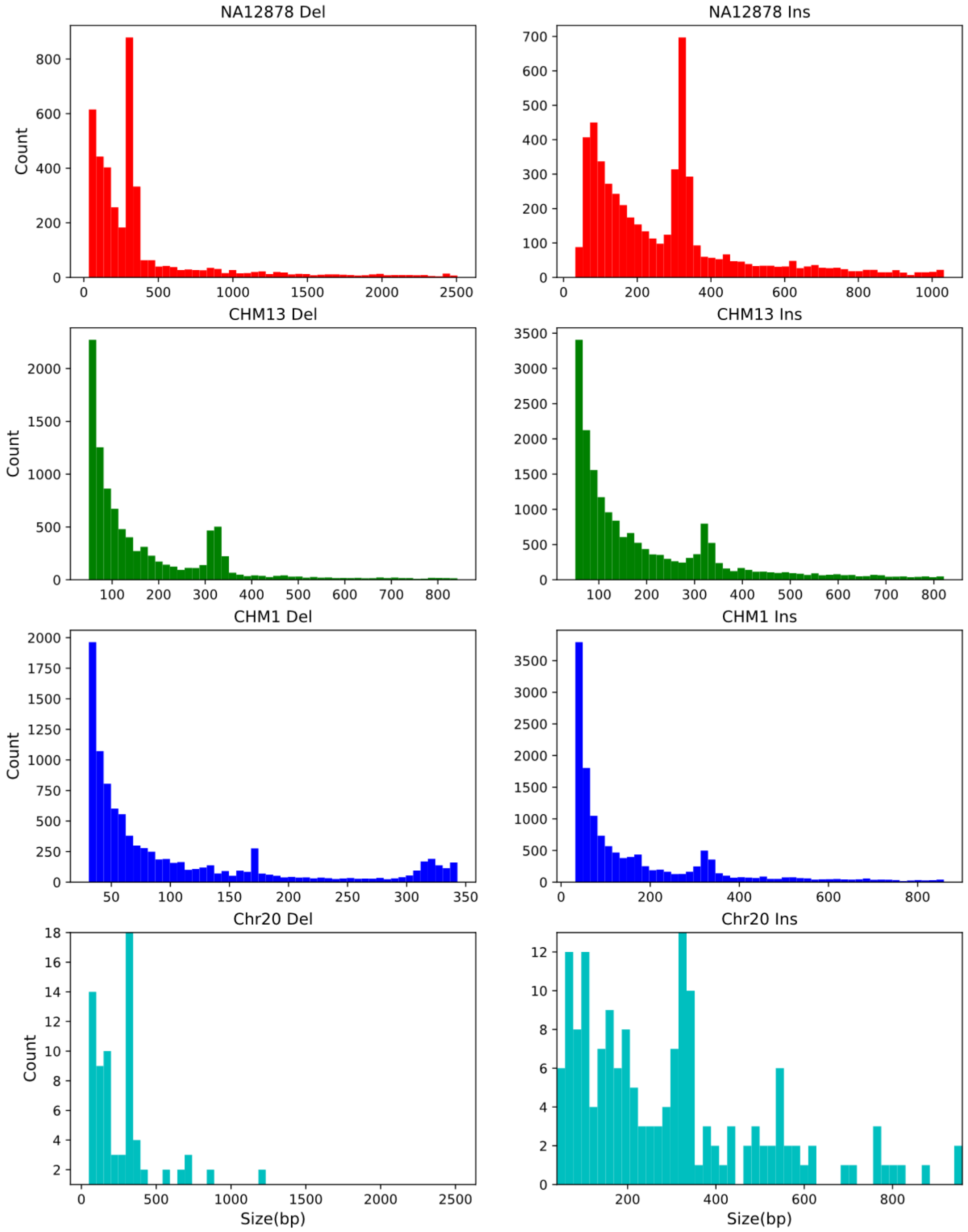

Fig. S2: Quality of each SV call set by size. **A)** deletions; **B)** insertions. In each size bin, the calls are divided into True positives (blue), False negatives (orange), and False positives (green), based on the comparison with the true set. Only SVs smaller than <1,000 bps are shown to improve visibility.

A)

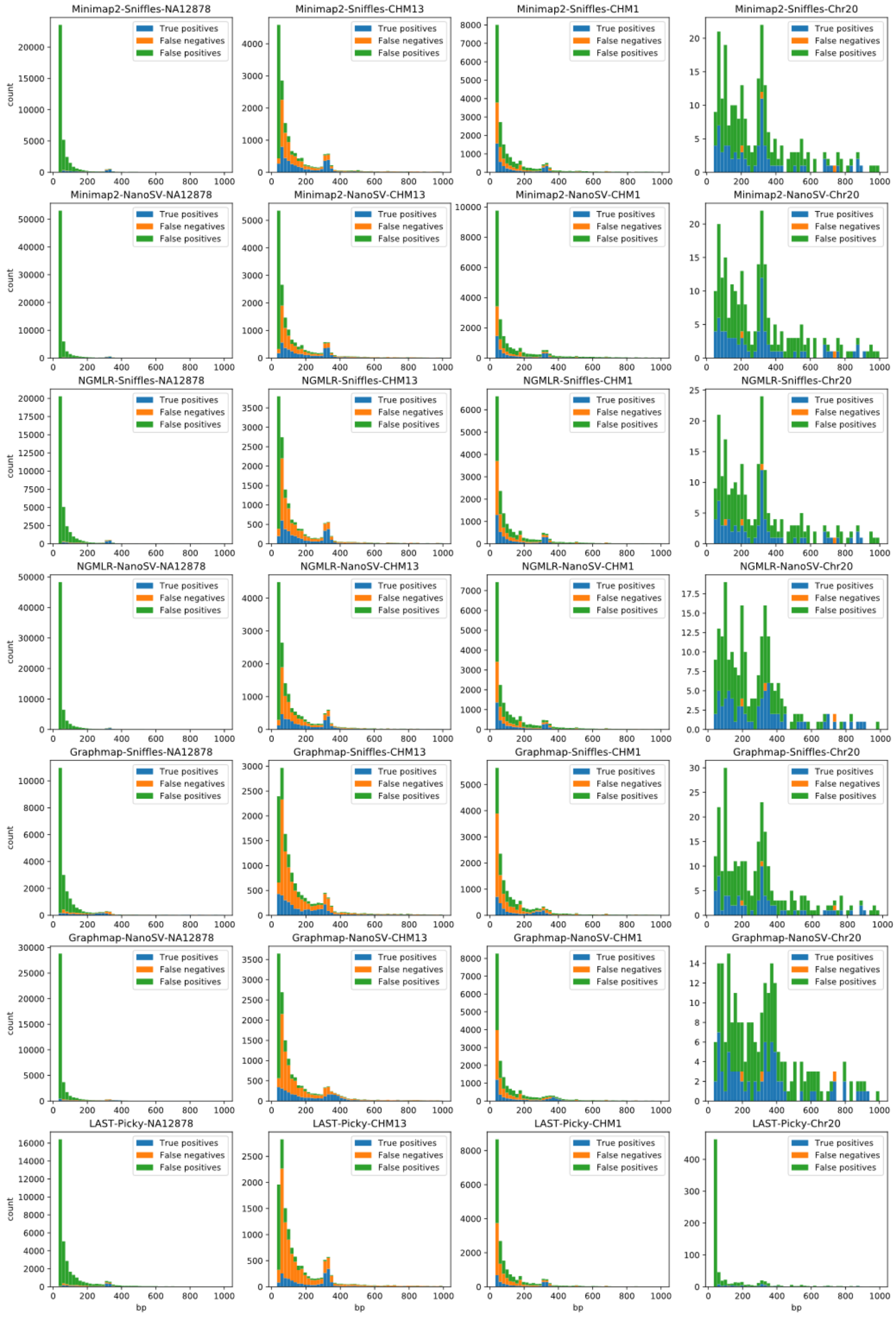

B)

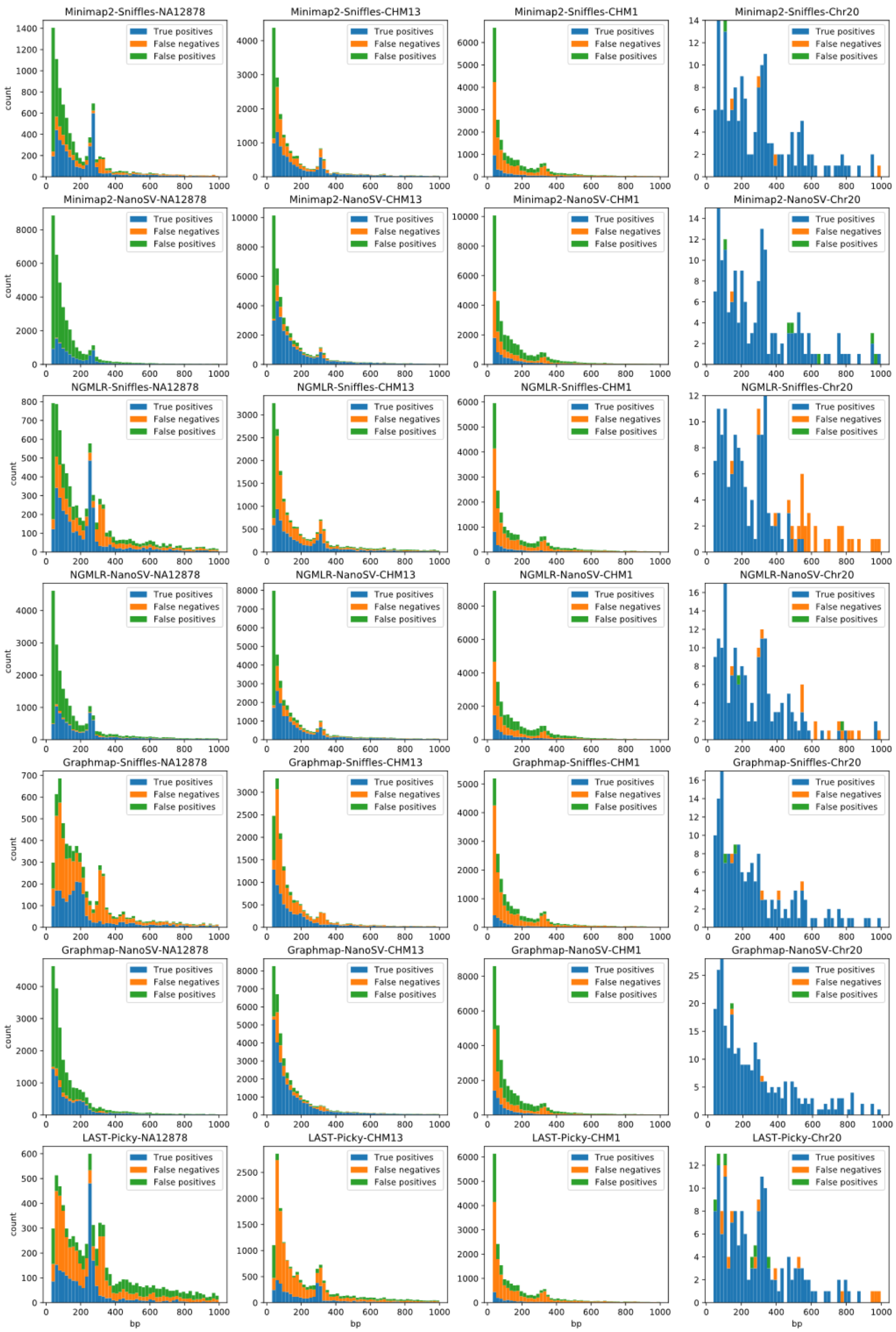
Minimap2-Sniffles-NA12878
Minimap2-Sniffles-CHM13
Minimap2-Sniffles-CHM1
Minimap2-Sniffles-Chr20
Minimap2-NanoSV-NA12878
Minimap2-NanoSV-CHM13
Minimap2-NanoSV-CHM1
Minimap2-NanoSV-Chr20
NGMLR-Sniffles-NA12878
NGMLR-Sniffles-CHM13
NGMLR-Sniffles-CHM1
NGMLR-Sniffles-Chr20
NGMLR-NanoSV-NA12878
NGMLR-NanoSV-CHM13
NGMLR-NanoSV-CHM1
NGMLR-NanoSV-Chr20
Graphmap-Sniffles-NA12878
Graphmap-Sniffles-CHM13
Graphmap-Sniffles-CHM1
Graphmap-Sniffles-Chr20
Graphmap-NanoSV-NA12878
Graphmap-NanoSV-CHM13
Graphmap-NanoSV-CHM1
Graphmap-NanoSV-Chr20
LAST-Picky-NA12878
LAST-Picky-CHM13
LAST-Picky-CHM1
LAST-Picky-Chr20
True positives
False negatives
False positives
count
bp

Fig. S3: Precision-recall graph of NA12878 SV calls before and after filtering repetitive genomic regions. SV call sets were filtered to exclude SVs overlapping "Simple_repeat" or "Low_complexity" regions in the human genome version hg38 based on RepeatMasker (http://www.repeatmasker.org/genomes/hg38/RepeatMasker-rm405-db20140131/hg38.fa.out.gz). Pipelines are represented with shapes and datasets are represented with colors.

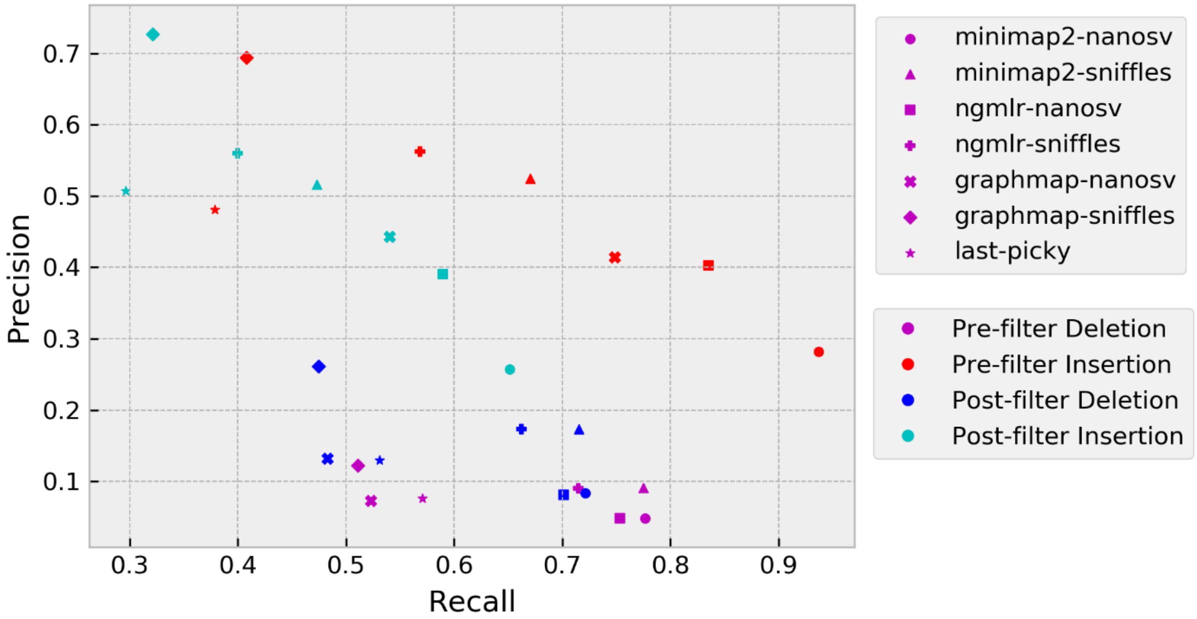

Fig. S4: SV call differences between pipelines. A) same mapper different callers; **B)** different mappers same caller; **C)** different mappers different callers.

A)

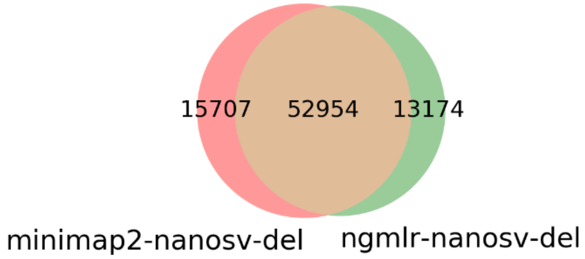

B)

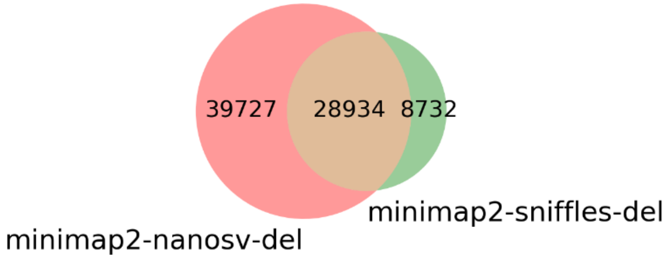

C)

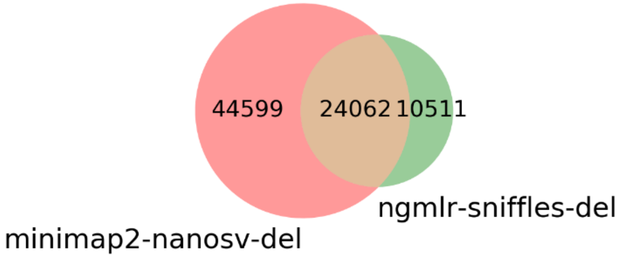

Fig. S5: Nanopore and PacBio sequencing alignments comparison at an SV region. IGV plot of an NA12878 SV region (chr1:117,029,131 - 117,029,278, red box) that was identified by all seven pipelines but absent in the true set. Top: SV call diagram of the seven pipelines; middle: nanopore sequence minimap2 alignments; bottom: PacBio sequence alignment.

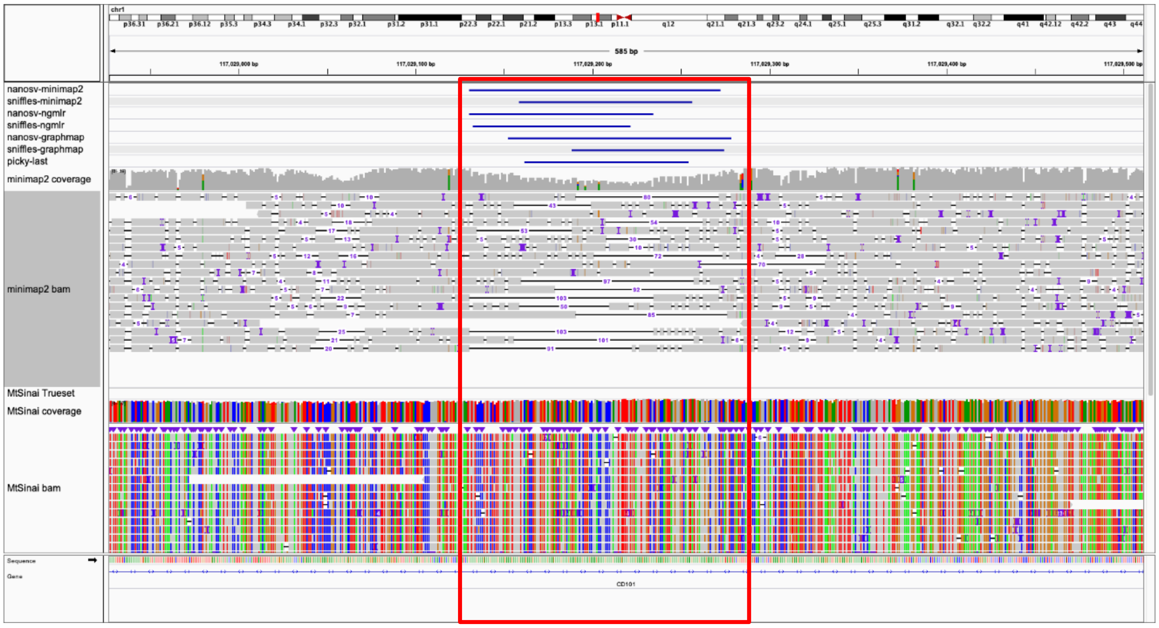

Table S1: SV call set evaluation.

	No. of SVs	Recall	Precision	F1 Score	1-99bp	100-499bp	500-999bp	1-5kb	5-10kb	>=10kb
**NA12878-Deletion**										
minimap2-nanosv	68,661	72.13%	8.34%	0.15	62,144	5,482	361	485	134	55
minimap2-sniffles	37,666	71.55%	17.32%	0.28	31,979	4,763	316	427	139	42
ngmlr-nanosv	66,128	70.11%	8.08%	0.14	58,596	6,387	398	534	158	55
ngmlr-sniffles	34,573	66.25%	17.34%	0.27	28,439	5,158	342	451	137	46
graphmap-nanosv	38,683	48.28%	13.25%	0.21	34,408	3,664	229	336	46	0
graphmap-sniffles	20,444	47.45%	26.15%	0.34	15,966	3,735	315	393	35	0
last-picky	33,814	53.13%	13.00%	0.21	24,889	7,599	854	342	108	22
true set	4,352				771	2,489	311	557	172	52
**CHM13-Deletion**										
minimap2-nanosv	12,155	50.25%	37.47%	0.43	7,572	3,589	383	450	99	62
minimap2-sniffles	11,147	46.81%	44.19%	0.45	6,874	3,271	376	456	109	61
ngmlr-nanosv	11,060	49.95%	39.33%	0.44	6,586	3,394	396	518	109	57
ngmlr-sniffles	9,424	44.97%	46.52%	0.46	5,627	2,765	340	501	121	70
graphmap-nanosv	8,627	36.00%	44.38%	0.40	5,015	2,697	293	474	105	43
graphmap-sniffles	8,155	32.16%	47.74%	0.38	4,172	2,895	359	556	121	52
last-picky	6,119	26.48%	42.93%	0.33	3,192	2,194	252	374	90	17
true set	10,671				4,490	4,736	486	750	166	43
**CHM1-Deletion**										
minimap2-nanosv	18,641	40.96%	25.12%	0.31	11,249	5,438	1,099	726	96	33

minimap2-sniffles	14,741	34.83%	30.88%	0.33	9,280	4,494	549	341	55	22
ngmlr-nanosv	14,600	39.20%	29.77%	0.34	8,357	4,977	679	489	68	30
ngmlr-sniffles	12,005	34.62%	35.02%	0.35	7,103	3,934	499	382	60	27
graphmap-nanosv	12,998	28.83%	30.18%	0.29	7,985	4,243	473	285	12	0
graphmap-sniffles	8,954	22.77%	34.71%	0.27	4,806	3,533	351	256	8	0
last-picky	13,089	24.10%	21.06%	0.22	8,576	3,752	395	302	47	17
true set	10,784				6,581	3,457	317	329	89	11
**Chr20-Deletion**										
minimap2-nanosv	279	97.92%	33.69%	0.50	42	156	42	31	7	1
minimap2-sniffles	275	96.88%	33.82%	0.50	46	153	39	30	5	2
ngmlr-nanosv	238	96.88%	39.08%	0.56	43	151	23	16	4	1
ngmlr-sniffles	272	95.83%	33.95%	0.50	44	151	39	31	4	3
graphmap-nanosv	270	94.79%	33.70%	0.50	35	159	41	32	3	0
graphmap-sniffles	313	92.71%	28.62%	0.44	57	181	42	31	2	0
last-picky	790	92.71%	11.37%	0.20	540	170	42	30	5	3
true set	96				14	49	13	15	4	1
**NA12878-Insertion**										
minimap2-nanosv	39,260	65.12%	25.71%	0.37	22,024	14,709	1,208	1,117	155	47
minimap2-sniffles	7,428	47.29%	51.62%	0.49	3,403	3,474	341	192	13	5
ngmlr-nanosv	21,971	58.95%	39.06%	0.47	10,389	8,974	1,436	1,047	114	11
ngmlr-sniffles	5,860	39.96%	56.03%	0.47	2,025	2,957	589	235	37	17
graphmap-nanosv	22,046	54.00%	44.29%	0.49	11,682	8,452	1,050	847	15	0
graphmap-sniffles	3,426	32.11%	72.73%	0.45	885	1,918	324	299	0	0

last-picky	4,574	29.64%	50.77%	0.37	703	2,547	852	460	9	3
true set	5,783				1,089	3,461	622	570	28	13
**CHM13-Insertion**										
minimap2-nanosv	25,885	64.71%	63.80%	0.64	12,722	11,320	1,008	759	70	6
minimap2-sniffles	10,989	55.92%	64.52%	0.60	5,526	4,689	492	264	16	2
ngmlr-nanosv	20,617	49.00%	60.58%	0.54	9,665	9,552	937	421	40	2
ngmlr-sniffles	9,915	41.79%	61.69%	0.50	4,496	4,449	648	316	2	4
graphmap-nanosv	22,068	57.13%	69.09%	0.63	11,746	9,147	738	437	0	0
graphmap-sniffles	7,167	41.93%	71.39%	0.53	3,364	3,266	313	224	0	0
last-picky	7,952	20.88%	55.66%	0.30	3,977	3,464	370	139	1	1
true set	15,158				7,426	5,275	1,118	1,157	148	34
**CHM1-Insertion**										
minimap2-nanosv	194	12.88%	22.65%	0.16	34	108	32	18	2	0
minimap2-sniffles	175	11.12%	25.43%	0.15	28	106	27	14	0	0
ngmlr-nanosv	171	11.55%	23.46%	0.15	37	117	15	2	0	0
ngmlr-sniffles	133	10.13%	25.95%	0.15	29	101	2	0	0	1
graphmap-nanosv	303	9.85%	23.83%	0.14	81	168	40	14	0	0
graphmap-sniffles	179	7.26%	26.97%	0.11	44	98	25	12	0	0
last-picky	158	6.64%	22.70%	0.10	30	104	23	0	0	1
true set	181				29	106	29	15	1	1
**Chr20-Insertion**										
minimap2-nanosv	40,536	90.61%	94.94%	0.93	20,918	15,644	2,044	1,623	196	111
minimap2-sniffles	14,421	88.95%	99.38%	0.94	7,298	5,145	946	869	121	42
ngmlr-nanosv	28,426	76.24%	99.35%	0.86	14,272	10,358	1,916	1,585	216	79
ngmlr-sniffles	11,960	66.85%	99.18%	0.80	5,244	4,344	1,194	1,033	103	42

graphmap-nanosv	35,734	90.06%	99.64%	0.95	18,142	13,193	1,997	2,062	302	38
graphmap-sniffles	10,006	88.95%	98.80%	0.94	4,639	3,713	686	811	130	27
last-picky	6,974	76.80%	97.20%	0.86	1,976	3,134	1,082	743	30	9
true set	14,779				5,061	6,848	1,354	1,292	178	46

Table S2: Aligners and SV callers excluded from the analysis.

Name	Type	Version	Release Year	Language	Description	Citation
Meta-aligner	Aligner	N/A	2017	C++	Run failed multiple times	[1]
MashMap	Aligner	2	2017	C++	Only aligns long reads	[2]
BLASR	Aligner	5.3.2	2012	C++	Only compatible with PacBio reads	[3]
SMRT-SV	SV Caller	N/A	2017	Python	Requires BLASR output	[4]
HySA	SV Caller	N/A	2017	Perl	Requires additional Illumina reads	[5]
PBHoney	SV Caller	15.8.24	2014	Python	Only compatible with PacBio reads	[6]

Table S3: Counts of different types of NA12878 SVs called by the seven pipelines.

SV Type	minimap2-sniffles	minimap2-nanosv	ngmlr-sniffles	ngmlr-nanosv	graphmap-sniffles	graphmap-nanosv	last-picky
**DEL**	38,364	82,989	34,877	82,175	21,166	52,019	41,525
**DUP**	111	491	634	523	0	0	2,535
**INS**	7,645	39,698	5,377	22,096	3,629	22,289	2,102
**Others**	280	0	280	0	29	0	19
DEL: deletion; DUP: duplication; INS: insertion; Others: inversion, translocation, etc.							

Table S4: Statistics of random forest classifier on all datasets.

Dataset	Accuracy	Recall	Precision	F1 score	Feature contribution						
					SVLEN	MAPQ	CIPOS	CIEND	PRECISE	RE	DEPTHPVAL
**CHM13-Deletion**	0.78	0.83	0.75	0.79	52.13%	10.08%	1.80%	2.91%	5.37%	7.31%	20.40%
**CHM13-Insertion**	0.74	0.92	0.73	0.82	55.40%	8.47%	2.42%	3.03%	1.32%	14.41%	14.95%
**NA12878-Deletion**	0.94	0.59	0.69	0.64	62.78%	1.89%	13.54%	6.98%	1.73%	1.84%	11.24%
**NA12878-Insertion**	0.69	0.48	0.64	0.55	41.90%	3.78%	20.23%	25.35%	3.26%	5.48%	0.00%
**CHM1-Deletion**	0.76	0.19	0.74	0.31	19.58%	4.78%	15.80%	6.78%	5.19%	12.65%	35.22%
**CHM1-Insertion**	0.75	0	N/A	N/A	39.96%	20.36%	6.12%	15.76%	3.51%	10.78%	3.51%
**Chr20-Deletion**	0.77	0.36	0.66	0.47	13.86%	4.07%	9.84%	9.09%	1.15%	13.40%	48.58%
**Chr20-Insertion**	0.98	1.00	0.98	0.99	11.79%	60.37%	3.75%	19.57%	0.00%	4.52%	0.00%

N/A: undefined metrics due to no predicted true samples.

References

1. Nashta-ali D, Aliyari A, Ahmadian Moghadam A, Edrisi MA, Motahari SA, Hossein Khalaj B: **Meta-aligner: long-read alignment based on genome statistics.** *BMC Bioinformatics* 2017, **18:**126-126.
2. Jain C, Dilthey A, Koren S, Aluru S, Phillippy AM: **A Fast Approximate Algorithm for Mapping Long Reads to Large Reference Databases.** In: Springer, Cham; 2017: 66-81
3. Chaisson MJ, Tesler G: **Mapping single molecule sequencing reads using basic local alignment with successive refinement (BLASR): application and theory.** *Bmc Bioinformatics* 2012, **13**.
4. Huddleston J, Chaisson MJP, Steinberg KM, Warren W, Hoekzema K, Gordon D, Graves-Lindsay TA, Munson KM, Kronenberg ZN, Vives L, et al: **Discovery and genotyping of structural variation from long-read haploid genome sequence data.** *Genome Research* 2017, **27:**677-685.
5. Fan X, Chaisson M, Nakhleh L, Chen K: **HySA: a Hybrid Structural variant Assembly approach using next-generation and single-molecule sequencing technologies.** *Genome research* 2017, **27:**793-800.
6. English AC, Salerno WJ, Reid JG: **PBHoney: identifying genomic variants via long-read discordance and interrupted mapping.** *BMC Bioinformatics* 2014, **15:**180.
