# Supplementary material for: Evaluating nanopore sequencing data processing pipelines for structural variation identification
Source: Genome Biol. 2019 Nov 14;20:237. doi: 10.1186/s13059-019-1858-1 (PMC6857234; doi:10.1186/s13059-019-1858-1)
Supplement: Supplementary file 2 — Additional file 2. Review history. [file 13059_2019_1858_MOESM2_ESM.docx]

Review History

**First round of review**

**Reviewer 1**

**Are you able to assess all statistics in the manuscript, including the appropriateness of statistical tests used?**

Yes, and I have assessed the statistics in my report.

**Comments to author:**

This study evaluated aligners and callers for SV identification from nanopore data. They used three human nanopore datasets, including both real and simulated data. Overall, I think this paper is not significant enough to be published on Genome Biology. If the author could propose a new method and benchmarked with other methods, or they could choose the best tool to analyze data and get new insights, this work would be a better fit. It is known that different aligners and callers generate very different results and low coverage libraries lead to bad calls. The authors just observed it again and did not provide new insights to mitigate these problems. The suggestion to simply combine results from multiple pipelines is not appropriate. If the authors can make all their pipelines available, it will make better impact.      

Major comments: 
1. In Figure 3, the recall and precision for Chr20 data, especially insertions behaved very different from two real datasets. Does this mean the simulation itself is not reasonable enough? 
2. It is not clear what do the output of different methods look like. Do they report magnitude and significant level of SV calls? 
3. The author uses F1 score to justify the decision to integrate results from multiple pipelines. Though true and false positive numbers are important, but it is more appropriate to evaluate false discovery rate. When combing results from multiple methods, how to rank their significance? This determines how we adjust for multiple testing and calculate false discovery rate. Instead of naive integration of results from multiple methods, statistically it is more appropriate to integrate summary statistics through Fisher's omnibus method. But because the authors do not describe the output from different callers in details, I don't know the nature of those callers. I can't give more useful suggestions based on the current version.   
4. On their GitHub page, all codes are organized as separated shell files. I would suggest the authors to make their recommended pipelines using things like Snakemake so that other people could directly use the pipelines.

**Reviewer 2**

**Are you able to assess all statistics in the manuscript, including the appropriateness of statistical tests used?**

Yes, and I have assessed the statistics in my report.

**Comments to author:**

This paper reports on accuracy and computational requirement benchmarks of several read mappers and structural variation (SV) discovery tools using nanopore sequencing data. The paper overall is well planned and executed. One major obstacle, which is not caused by the authors, is that there are only a very limited number of read mappers and SV callers using nanopore, and even fewer WGS data sets generated using nanopore. Obviously, I am not expecting the authors to "solve" this problem, just pointing out in case there are concerns about the low number of tools benchmarked in this manuscript.


The paper can still be improved:

1- Although the tools tested here claim to be "SV callers", most tests are done on large insertions and deletions. "Structural variation" implies other types of variants. This can be just due to the limitations of the tools tested here, but some of them can call inversions for example (also as seen in Sup Fig 2). Some results on the tools that can call inversions, duplications, translocations, or other forms of SV would be beneficial. Otherwise maybe most of the paper should be edited to switch from "SV" to "large indels".

2- As I mentioned above, there aren't many available data sets, therefore the authors used NA12878 as the real data, and simulated reads from CHM1 assembly in addition to a full-simulation using chromosome 20. One other data set that would be useful here is using the CHM13 nanopore WGS data released by the same consortium that generated NA12878 (https://github.com/nanopore-wgs-consortium/CHM13). This data set has multiple benefits: newer than NA12878, there are orthogonal call sets generated with other data, and there is even a telomere-to-telomere assembly to compare against.

3- I appreciate that the authors released all their scripts. It would also be very good for the community if they can release the call sets generated by mapper-caller pairs for all data sets in addition to the full data for chromosome 20 simulation. This can be done through an archival site such as Zenodo.

4- Figure legends are too short and not descriptive enough. Ideally they should be comprehensive enough to minimize to go and hunt for more explanation in the main text.

5- Main paper figures are very low resolution in the PDF, which makes in hard to read.

6- Not sure if this is a resolution issue, but the "truth" for NA12878 deletions in Fig 2 looks too close to 0. This is more likely due to scale, where some callers have 60000 deletion calls, and the truth is likely around 800-900. This figure can be replaced with a table, I find tables more informative than figures almost always anyway. The table can also include sensitivity and specificity numbers, to keep all necessary information together.


Minor:

- Page9: "Sniffle" -> "Sniffles"
- Supplementary Figure 2: the "others" section looks empty. Zoom on PDF shows there are two data points there, but again the readability is low. Maybe it can be replaced by a table; or a number on top of the "unseen" data points can be added.

**Authors Response**

**Point-by-point responses to the reviewers’ comments:**

Comment

*Response*

Review 1

Referee 1 raises serious concerns that the current manuscript lacks sufficient new insights or a useful guideline for the end users.

- *We understand Referee 1’s concern regarding new insights and guidelines. We addressed the issues in several ways:*

*To improve the novelty of the study and usefulness of our analysis pipeline, we now included a machine learning approach based on the random forest algorithm to integrate call sets from different pipelines. The approach uses seven features in the output of different SV callers to train a classifier based on the true set. We showed that the integrated call set has substantially higher sensitivity and accuracy compared to individual call sets.*

*To improve the usefulness of our analysis, we integrated our analyses into flexible pipelines and provide all the tools and pipelines used in the analysis, including the integration program, as a singularity package (available at* [*https://github.com/JXing-Lab/nanopore-sv-evaluation/tree/master/singularity*](https://github.com/JXing-Lab/nanopore-sv-evaluation/tree/master/singularity)*). The pipelines, integration programs, and the singularity package will help other researchers to perform the analysis described in this study in an efficient way.*

*We agree with the reviewer that it is important to provide the output files of different tools. We now provided output files from all pipelines as part of the revision (available at* [*https://doi.org/doi:10.7282/t3-zw94-js46*](https://and) *and* [*https://github.com/JXing-Lab/nanopore-sv-evaluation/release*](https://github.com/JXing-Lab/nanopore-sv-evaluation/releases)*s).*

1. In Figure 3, the recall and precision for Chr20 data, especially insertions behaved very different from two real datasets. Does this mean the simulation itself is not reasonable enough?

-	*The simulation was generated based on the read profile of a real nanopore run and the SV calls were from a real human SV call set (NA12878). Therefore, we think the simulation itself is reasonable. We believe the difference in recall and precision for the Chr20 dataset are mainly caused by the nature of the true sets used for evaluation of the pipelines. For the Chr20 dataset we used a pre-defined set of SVs. In contrary, the other three datasets used SV true sets derived from the PacBio sequencing platform. As we showed in the Supplemental Figure 5, it is possible that some of the SV calls missing in the true set are due to the differences between the Nanopore and PacBio sequencing platforms. Besides the true set differences, we agree that the simulated Chr20 dataset contains only a single human chromosome, so it might not fully reflect all the heterogeneity of the human genome. We addressed this point in the discussion (p15):*

*“The advantage of such simulated dataset is that we know precisely the true SVs that can later be used to evaluate different pipelines. Nevertheless, the simulated reads are based solely on the chromosome 20 and are unlikely to capture the true heterogeneity of the entire human genome.”*

It is not clear what do the output of different methods look like. Do they report magnitude and significant level of SV calls?

-	*We agree with the reviewer that it is important to provide the output files of different tools. We have uploaded the final SV call sets obtained in this study as a supplemental data which is now available at* [*https://doi.org/doi:10.7282/t3-zw94-js46*](https://and) *and* [*https://github.com/JXing-Lab/nanopore-sv-evaluation/releases*](https://github.com/JXing-Lab/nanopore-sv-evaluation/releases)*.*

*-	In term of significance, no caller provides an overall p-value for each variant. Only NanoSV provides a p-value for the depth of coverage at the breakpoints.*

The author uses F1 score to justify the decision to integrate results from multiple pipelines. Though true and false positive numbers are important, but it is more appropriate to evaluate false discovery rate. When combing results from multiple methods, how to rank their significance? This determines how we adjust for multiple testing and calculate false discovery rate. Instead of naive integration of results from multiple methods, statistically it is more appropriate to integrate summary statistics through Fisher's omnibus method. But because the authors do not describe the output from different callers in details, I don't know the nature of those callers. I can't give more useful suggestions based on the current version.

- *We appreciate the reviewer’s suggestion on integrating call sets and agree the Fisher’s omnibus method would be a better way to integrate the call sets, provided that the p-values are available. Unfortunately, the three SV callers we used do not provide p-values for the reported SVs. Because it is difficult to determine the relative contributions of different quality measures provided by different SV callers, we adopted a machine learning approach based on the random forest algorithm. The algorithm takes into account seven features outputted by the SV callers as training features (Table 4), and produces an integrated call set. We showed that the integrated call set obtained from this machine learning algorithm has substantially higher sensitivity and accuracy compared to individual call sets and the simple consensus approach (Figure 6B).*

*-	As reviewer suggested, to assess the false discovery rate of the two integration*

*methods we explored, we now included a precision-recall plot (Figure 6B).*

On their GitHub page, all codes are organized as separated shell files. I would suggest the authors to make their recommended pipelines using things like Snakemake so that other people could directly use the pipelines.

-	*As the reviewer suggested, we integrated our analyses into flexible pipelines and provide all the programs and pipelines used in the analysis as a singularity package (available at* [*https://github.com/JXing-Lab/nanopore-sv-evaluation/tree/master/singularity*](https://github.com/JXing-Lab/nanopore-sv-evaluation/tree/master/singularity)*). The pipelines, integration programs, and the singularity package will help other researchers to use the pipelines described in this study efficiently.*

Review 2

Although the tools tested here claim to be "SV callers", most tests are done on large insertions and deletions. "Structural variation" implies other types of variants. This can be just due to the limitations of the tools tested here, but some of them can call inversions for example (also as seen in Sup Fig 2). Some results on the tools that can call inversions, duplications, translocations, or other forms of SV would be beneficial. Otherwise maybe most of the paper should be edited to switch from "SV" to "large indels".

-	*We agree with the reviewer that the evaluation is limited to insertions and deletions. Because some of the true sets only contain insertions and deletions, it is difficult to provide evaluations of other types of SVs. As the reviewer suggested, we revised the relevant part of paper and changed “SVs” to “indels”.*

As I mentioned above, there aren't many available data sets, therefore the authors used NA12878 as the real data, and simulated reads from CHM1 assembly in addition to a full-simulation using chromosome 20. One other data set that would be useful here is using the CHM13 nanopore WGS data released by the same consortium that generated NA12878. This data set has multiple benefits: newer than NA12878, there are orthogonal call sets generated with other data, and there is even a telomere-to-telomere assembly to compare against.

- *We appreciate the reviewer’s suggestion of using the new CHM13 dataset. As suggested, we added the CHM13 dataset to our analyses. The CHM13 showed better performance than NA12878, likely due to a higher quality reference call set and higher sequencing coverage. We now report results from the CHM13 genome analyses, and used CHM13 results as the training set for the machine learning-based integration approach.*

I appreciate that the authors released all their scripts. It would also be very good for the community if they can release the call sets generated by mapper-caller pairs for all data sets in addition to the full data for chromosome 20 simulation. This can be done through an archival site such as Zenodo.

-	*We agree with the reviewer that it is important to provide the output files of different tools. We have uploaded the final SV calls as supplemental data (available at* [*https://doi.org/doi:10.7282/t3-zw94-js46*](https://and) *and* [*https://github.com/JXing-Lab/nanopore-sv-evaluation/release*](https://github.com/JXing-Lab/nanopore-sv-evaluation/releases)*s).*

*-	In addition, we integrated our analyses into flexible pipelines and provide all the tools used in the analysis, including the integration program, as a singularity package (available at* [*https://github.com/JXing-Lab/nanopore-sv-evaluation/tree/master/singularity*](https://github.com/JXing-Lab/nanopore-sv-evaluation/tree/master/singularity)*). The pipelines, integration programs, and the singularity package will help other researchers to perform the analysis described in this study in their own analyses.*

Figure legends are too short and not descriptive enough. Ideally they should be comprehensive enough to minimize to go and hunt for more explanation in the main text.

-	*We have edited the figure legends to include more explanations.*

Main paper figures are very low resolution in the PDF, which makes in hard to read.

-	*We have generated the figures at a higher resolution in this revised version of the manuscript.*

Not sure if this is a resolution issue, but the "truth" for NA12878 deletions in Fig 2 looks too close to 0. This is more likely due to scale, where some callers have 60000 deletion calls, and the truth is likely around 800-900. This figure can be replaced with a table, I find tables more informative than figures almost always anyway. The table can also include sensitivity and specificity numbers, to keep all necessary information together.

-	*We agree with the reviewer that some of the columns in Figure 2 are difficult to read. We have included the counts of each bin in the Supplemental Table 1.*

Page9: "Sniffle" -> "Sniffles"
- *We corrected the typo.*

Supplementary Figure 2: the "others" section looks empty. Zoom on PDF shows there are two data points there, but again the readability is low. Maybe it can be replaced by a table; or a number on top of the "unseen" data points can be added.

-	*We agree with the reviewer and have replaced Supplementary Figure 2 with Supplementary Table 3.*

**Second round of review**

**Reviewer 1**

This work has been significantly improved. In the revision, the authors proposed a random forest approach to integrate SV calls from different origins to get a more confident set. They showed the performance of this approach on Figure 5, but only for CHM13 dataset. I wonder whether the authors can add such comparison for all previous datasets to make sure the conclusion is general.

**Reviewer 2**

The authors improved their manuscript based on both reviewers' comments. I also appreciate Supplementary Table 1; but I request the authors to add the "difficult to see" numbers on top of Figure 2 (similar to https://web.mit.edu/course/21/21.guide/patents.gif)

**Authors Response**

**Point-by-point responses to the reviewers’ comments:**

Reviewer 1

This work has been significantly improved. In the revision, the authors proposed a random forest approach to integrate SV calls from different origins to get a more confident set. They showed the performance of this approach on Figure 5, but only for CHM13 dataset. I wonder whether the authors can add such comparison for all previous datasets to make sure the conclusion is general.

*As the reviewer suggested, we included the performance of the random forest classifier integration method for all datasets in Table S4. Overall, the classifier performed consistently across all data sets (~75% accuracy on average) and provided improvement over individual call set. For the CHM1 Insertion data set, the labeled data set has an imbalanced class distribution (~75% false vs ~25% true). Therefore, the algorithm made no positive prediction. This result highlights the need of a high-quality true set for the machine learning algorithm.*

Reviewer 2

The authors improved their manuscript based on both reviewers' comments. I also appreciate Supplementary Table 1; but I request the authors to add the "difficult to see" numbers on top of Figure 2 (similar to https://web.mit.edu/course/21/21.guide/patents.gif)

*As the reviewer suggested, we updated Figure 2 and added numbers on top of the bars that are below the first tick.*
